# The influence of light on Interleukin-10: A preliminary study

**DOI:** 10.1016/j.bbih.2024.100887

**Published:** 2024-10-06

**Authors:** Liza Mekschrat, Michael Göring, Bjarne Schmalbach, Nicolas Rohleder, Katja Petrowski

**Affiliations:** a Medical Psychology & Medical Sociology, University Medical Center of the Johannes Gutenberg University Mainz, Mainz, Germany; b Chair of Health Psychology, Department of Psychology, Friedrich-Alexander-Universität Erlangen-Nürnberg, Erlangen, Germany

**Keywords:** Circadian rhythms, Light irradiation, IL-10, Cortisol awakening response

## Abstract

Light influences circadian rhythms, including that of the stress hormone cortisol. Cortisol, in turn, has been observed to promote expression of the anti-inflammatory cytokine IL-10. It is thus of interest whether the cytokine IL-10 is also influenced by light, perhaps in accord with the diurnal variations in cortisol. Hence, this highly standardized preliminary sleep laboratory study in healthy adult men investigated a potential influence of different light exposure on IL-10 and cortisol concentrations in blood. In a between-subject design, *N* = 42 participants were exposed to either bright, dim, blue or red light after wake-up. Two mixed-model analyses with the factors of light condition and time (across eight IL-10 and cortisol sampling points) were conducted. Additionally, area under the curve measurements (AUCg and AUCi) were calculated for both cortisol and IL-10. Across all conditions, IL-10 and cortisol concentrations significantly changed over time. However, none of the light conditions exerted a greater influence on IL-10 or cortisol levels than others. For cortisol, there was greater total output (AUCg) in the blue-light condition in particular. Further research is needed to gain insight into whether or not types of light or cortisol levels have a hand in influencing natural IL-10 concentrations.

## 1 Introduction

Light is an important driver of circadian rhythmicity (e.g., LeGates et al., 2014), i.e., the entrainment of biological processes to the 24-h day and night cycle (Takahashi, 2017). Distinct circadian rhythms can be observed, amongst others, in the endocrine system (e.g., Oster et al., 2017). Notably, the hormone cortisol oscillates on a distinct rhythm, increasing in the first 30–40 min after wake-up, peaking thereupon (termed the cortisol awakening response or CAR, e.g., Stalder et al., 2016), and then ebbing over the course of the rest of the day (e.g., Logan and McClung, 2019; Stalder et al., 2016).

Light information is projected to the suprachiasmatic nucleus (SCN), the central circadian clock, via intrinsically photosensitive retinal ganglion cells (ip RGCs; LeGates et al., 2014). These ipRGCs express at 480 nm (nm), i.e., where light appears blueish to the eye (Lucas et al., 2014).

Evidence on the influence of light on cortisol has suggested that cortisol concentrations increase more strongly after exposure to bright light when compared to dim light (Petrowski et al., 2022), and blue light when compared to dim (Figueiro and Rea, 2012) or red light (Petrowski et al., 2019, 2020a) – although results on the effects of blue light remain inconclusive (Petrowski et al., 2020b). Glucocorticoids, amongst them cortisol, also play an important role in immune regulation, including inflammatory processes (Cain and Cidlowski, 2017; Silverman and Sternberg, 2012). They are widely understood to exert anti-inflammatory effects, inhibiting pro-inflammatory parameters (e.g., Cain and Cidlowski, 2017; Scheiermann et al., 2013). As such, cortisol has been observed to foster production of the anti-inflammatory cytokine Interleukin-10 (IL-10; Cain and Cidlowski 2017; del Giudice and Gangestad, 2018).

Much evidence has been collected in recent years on circadian rhythmicity governing immune processes (e.g., Haspel et al., 2020; Man et al., 2016; Scheiermann et al., 2013). At the same time, is has been noted that light can set the glucocorticoid-immune cycle in motion via HPA-axis activation (Cain and Cidlowski, 2017). If these mechanisms translate to light governing IL-10 levels remains, however, unclear. In healthy adults, there is some literature reporting circadian oscillations in IL-10, peaking during the day, potentially mediated by cortisol (Lange et al., 2010). Others found two peaks during the day (Young et al., 1995) or no indication of any clear rhythmicity along the day-and-night cycle (Lissoni et al., 1998). Regarding the effects of light on IL-10 concentrations, Feuerstein et al. (2011) found increased IL-10 levels in the cells of mice after blue-light exposure. In contrast, a recent study in post-operative appendicitis patients revealed reduced IL-10 concentrations after exposure to blue light when compared to standard hospital lighting (Lewis et al., 2018). To the knowledge of the authors, no research on the effects of different types of light on IL-10 concentrations in healthy adults has been conducted so far.

Therefore, the aim of this preliminary, but highly standardized sleep laboratory study, was to observe the effects of different types of light exposure (bright light, dim light, red light and blue light) on IL-10 concentrations in blood. Additionally, the aim was to replicate findings on the effects of these types of light irradiation on cortisol concentrations as well. As cortisol oscillations are particularly distinct and visible during the CAR and because any influence of exposure to light prior to the actual experimental light exposure was to be ruled out, the first 2 h after wake-up were chosen as the time frame for testing.

Hypothesis: Based on the reported results of the effects of light on cortisol as well as the influence of cortisol on IL-10, it was hypothesized that bright light and blue light exposure after wake-up would lead to increased cortisol and IL-10 concentrations when compared to dim light or exposure to red light.

## 2 Method

### 2.1 Participants

To exclude any potential bias introduced by the influence of sex hormones on cortisol expression (e.g., Juster et al., 2016), only men were asked to participate in this preliminary study. The blood serum samples of *N* = 42 healthy adult men are included in this study, *n* = 11 in the bright-light, *n* = 11 in the dim-light, *n* = 10 in the blue-light and *n* = 10 in the red-light condition. Participants were *M* = 25.4, *SD* = 4.82 years old, ranging from ages 18 to 35. All sociodemographic characteristics of the sample are depicted in Table 1. Exclusion criteria included colorblindness, acute and chronic illnesses like allergies, metabolic disorders, autoimmune diseases, coronary heart disease, blood disorders as well as psychological disorders. The latter were excluded based on replies to the Structured Clinical Interview for the DSM-V (SCID-5; First et al., 2015). Also excluded were users of psychoactive drugs, smokers of more than ten cigarettes per day and a BMI of >27 kg/m^2^. The Ethics Committee of the Medical Faculty of the Technical University of Dresden, Germany approved the study protocol (No #EK353092014) and it was conducted in accordance with the Declaration of Helsinki decided in 2013. Every participant provided written informed consent before participation.

**Table 1:** Sociodemographic characteristics of the sample.

	M	SD	Min	Max
Age – total sample	25.4	4.82	18	35
Age – bright-light conditio	24.8	5.62	19	35
Age – dim-light condition	25.6	6.09	18	35
Age – blue-light condition	25.0	4.08	19	34
Age – red-light condition	26.2	3.39	19	30

	*n*	%
Gender		
Male	42	100
Female	0	0
Nationality		
German	35	83.3
Other	7	16.7
Relationship status		
Single	18	42.9
Non-committed relationships	3	7.1
Committed relationship	21	50
Educational degree		
Studying/in training	18	42.9
Apprenticeship	6	14.3
Master craftsman's degree	1	2.4
University degree	10	23.8
No educational degree	7	16.7

### 2.2 Procedure

**Fig. 1:**
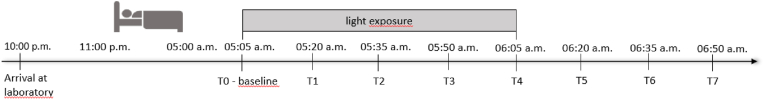
Study procedure on testing day.

The study was conducted in a between-subject design. Testing took place at the university's sleep laboratory. Upon arrival the night before testing (between 10:30–11:00 p.m.), the IV catheter used to collect IL-10 samples was placed. Participants went to bed at 11 p.m. and woken up at 5 a.m. the next morning. Sleep was monitored via actigraphy. Participants wore dark sunglasses when using the restrooms during the night or after wake-up. Five minutes after wake-up, the first blood sample was collected, followed by seven subsequent samples every 15 min. Light exposure commenced 10 min after wake-up, lasting 60 min. After light exposure, participants were sat in a dimly-lit room (<3 Luminance (lux)) for the last three blood samples (see Fig. 1).

### 2.3 Light conditions

Two half Ulbricht spheres, indirectly illuminated through LEDs distributed equally along the inside of the opening were used for light irradiation. To ensure homogeneous illumination of participants’ retinas, LEDs were covered with a spectral selective diffusor. Light in the different conditions was composited as follows: Blue light was 470–480 nm (nm) at 201 lux, red light was 635 nm at 235 lux, bright light (1241 lux) exposure was produced by simultaneously mixing blue, red and green (806 lux; peak wavelength 520 nm) light and dim light was white light at less than 3 lux.

Stray light levels in the testing were kept below 1 lux (at the eye) and lux were measured at eye-level with an illumination meter before and after each exposure. Sat in front of the Ulbricht spheres with their chins on a chinrest, eye-level was the same across all participants.

### 2.4 Biological markers: blood cytokines

The cortisol and IL-10 blood samples were collected with Serum-Gel-Monovette® (Sarstedt, Nümbrecht, Germany) and coagulated for 30 min at room temperature.

Subsequently, the samples were centrifuged at 20 °C and 2500xG RCF for 10 min. Before being assayed, the samples were stored at −80 °C. To determine cortisol concentrations, the Solid Phase Antigen Linked Technique (SPALT) was applied using a commercially available radioimmunoassay kit with the LIAISON-Analyzer® (DiaSorin, S. p.A., Italy). The lower detection limit was .43 nmol/l. The intra- and inter-assay coefficient of variation was <9.0 %. Cytokine concentrations were determined with highly-sensitive ELISA enzyme-linked immunosorbent assays (IBL International GmbH, Germany). The detection limit was <.04 pg/mL serum. The inter-assay coefficient was 10.1 %, the intra-assay coefficient was 8.7%.

### 2.5 Statistical analysis

Because of the highly-sensitive assays, a part of the IL-10 samples was below the detection limit. Of the *N* = 42 participants *n* = 5 had zero of the eight samples below the detection limit, *n* = 2 one sample, *n* = 12 two samples, *n* = 14 three samples and *n* = 9 four samples below the detection limit. Additionally, 14 of the 336 IL-10 samples (4.17%) in total were returned missing. Data sets with more than half of the IL-10 samples below the detection limit and/or missing were excluded beforehand and are not part of the *N* = 42 sample size. For the samples in question, a value of half of the detection limit (i.e., .02) was used for the analysis. As a result, IL-10 were not normally distributed and corrected using the natural logarithm ln.

T-Tests for independent samples were conducted to compare baseline cortisol concentrations between the different light conditions as well as baseline IL-10 concentrations between light conditions.

As some data points were returned missing by the laboratory as well, mixed model analyses wre chosen for the statistical analysis, as this provides greater reliability in datasets with missing values (Seltman, 2018). Subsequently, two mixed model analyses, one for IL-10 and one for cortisol concentrations, with the factors light (the different light conditions), time (the eight blood samples) and the interaction term of light and time were conducted in jamovi, using the module GAMLj. Additionally, area under the curve statistics with respect to ground (AUCg) and increase (AUCi) were calculated (Pruessner et al., 2003) and compared with t-tests for independent samples for both cortisol and IL-10 between each light condition.

## 3 Results

Table 2 provides mean IL-10 concentrations in each light condition for each measurement point. Table 3 lists mean cortisol concentrations for each measurement points across each light condition. Baseline IL-10 concentrations differed between the bright-vs. the dim-light condition, *t* (19) = −2.17, *p* = .043 and the bright-vs. red-light condition, *t* (19) = −2.16, *p* = .044. Baseline cortisol concentrations also did not differ significantly between most light conditions, except between the dim-vs. the blue-light condition, *t* (18) = −2.12, *p* = .048 and the blue-vs. the red-light condition, *t* (17) = 2.38, *p* = .029.

**Table 2:** Means, standard deviations and post-hoc comparisons for IL-10 concentrations per light condition and measurement point.

Measurement point (minutes after wake-up)	Bright light				Dim light				Blue light				Red light			
	*M*	*SD*	*p*_*uncorrected*_	*p*_*holm*_	*M*	*SD*	*p*_*uncorrected*_	*p*_*holm*_	*M*	*SD*	*p*_*uncorrected*_	*p*_*holm*_	*M*	*SD*	*p*_*uncorrected*_	*p*_*holm*_
T0 (+5)	.52	.64			1.41	1.19			.74	.80			1.25	.90		
T1 (+20)	1.86	1.31	.003∗	1.000	1.27	1.28	.973	1.000	.76	.73	.542	1.000	1.36	1.13	.964	1.000
T2 (+35)	1.55	1.23	.783	1.000	1.95	1.54	.336	1.000	.58	.52	.610	1.000	1.33	1.00	.921	1.000
T3 (+50)	1.31	.95	.645	1.000	1.43	1.30	.310	1.000	.89	.82	.437	1.000	1.45	.83	.606	1.000
T4 (+65)	1.69	1.38	.592	1.000	1.68	1.53	.869	1.000	2.31	2.51	.294	1.000	.96	.75	.239	1.000
T5 (+80)	1.70	1.44	.873	1.000	1.91	2.32	.796	1.000	2.24	1.77	.405	1.000	1.32	1.53	.765	1.000
T6 (+95)	1.31	1.64	.268	1.000	1.66	1.34	.461	1.000	1.56	1.57	.327	1.000	1.21	1.45	.767	1.000
T7 (+110)	.58	.69	.245	1.000	2.05	2.28	.826	1.000	.59	.91	.014∗	1.000	.68	.60	.483	1.000

*Note.* Means were logarithmised applying natural logarithm ln prior to and for mixed model analysis. *p-*values indicate mixed model post-hoc comparisons. ∗∗: *p* < .001. ∗: *p* < .05. Comparisons are to the proceeding time point each (T1 to T0, T2 to T1 etc.). All corrected comparisons apply Holm correction.

**Table 3:** Means, standard deviations and post-hoc comparisons for cortisol concentrations per light condition and measurement point.

Measurement point (minutes after wake-up)	Bright light				Dim light				Blue light				Red light			
	*M*	*SD*	*p*_*uncorr*_	*p*_*holm*_	*M*	*SD*	*p*_*uncorr*_	*p*_*holm*_	*M*	*SD*	*p*_*uncorr*_	*p*_*holm*_	*M*	*SD*	*p*_*uncorr*_	*p*_*holm*_
T0 (+5)	68.7	35.8			68.5	25.3			93	26.2			67.7	.90		
T1 (+20)	117	30.7	<.001∗∗	.002∗	125	28.1	<.001∗∗	<.001∗∗	153	33.9	<.001∗∗	<.001∗∗	128	1.13	<.001∗∗	<.001∗∗
T2 (+35)	158	27.6	<.001∗∗	.063	145	26.1	.049∗	1.000	165	35.4	.250	1.000	157	1.00	.008∗	1.000
T3 (+50)	164	18.7	.576	1.000	129	17	.124	1.000	165	47.7	.986	1.000	147	.83	.363	1.000
T4 (+65)	160	33.5	.759	1.000	116	20.5	.205	1.000	148	39	.103	1.000	135	.75	.269	1.000
T5 (+80)	141	26.6	.084	1.000	105	24.8	.302	1.000	139	47.8	.445	1.000	117	1.53	.096	1.000
T6 (+95)	127	50.4	.018∗	1.000	102	25.7	.717	1.000	129	45.7	.341	1.000	108	1.45	.419	1.000
T7 (+110)	115	42.1	.245	1.000	100	26.5	.915	1.000	105	38.5	.027∗	1.000	109	.60	.942	1.000

*Note. p-*values indicate mixed model post-hoc comparisons. ∗∗: *p* < .001. ∗: *p* < .05. Comparisons are to the proceeding time point each (T1 to T0, T2 to T1 etc.). *P*_*uncorr*_ = uncorrected *p*-values. All corrected comparisons apply Holm correction.

The mixed-model analysis for IL-10 returned a significant main effect for time, *F* (7, 250.6) = 2.36, *p* = .024. The main effect for light condition was not significant, *F* (3, 35.1) = .78, *p* = .511, neither was the interaction term, *F* (21, 250.6) = 1.35, *p* = .146. Although the main effect for time across all light condition was significant, Holm-corrected post-hoc comparisons did not reveal any significant changes from one measurement point to the next in each individual light condition.

Mean AUCg for IL-10 was *M* = 19.4, *SD* = 4.76 for the bright-light, *M* = 20.4, *SD* = 4.46 for the dim-light, *M* = 19.5, *SD* = 3.36 for the blue-light and *M* = 20.9, *SD* = 2.70 for the red-light condition. Mean AUCi for IL-10 was M = 6.18, SD = 6.10 for the bright-light, M = .54, SD = 7.71 for the dim-light, M = 2.91, SD = 8.20 for the blue-light and M = −2.96, SD = 6.24 for the red-light condition. AUCg for IL-10 was significantly different between the red- and the blue-light condition, *t* (151) = −3.37, *p* < .001, but not between any of the other conditions. AUCi for IL-10 proved significantly different between the bright- and the dim-light condition, *t* (174) = 5.14, *p* < .001, between the bright- and the blue-light condition, *t* (154) = 2.80, *p* = .006, between the bright- and the red-light condition, *t* (166) = 7.07, *p* < .001 and between the red- and the blue-light condition, *t* (147) = 3.36, *p* = .001.

The mixed-model analysis for cortisol revealed a significant main effect for time *F* (7, 261.2) = 49.12, *p* < .001, but none for light condition, *F* (3, 38.0) = 1.89, *p* = .147. The interaction term was not significant either, *F* (21, 261.2) = 1.50, *p* = .076.

Mean AUCg for cortisol was *M* = 909, *SD* = 206 for the bright-light, *M* = 807, *SD* = 86.5 for the dim-light, *M* = 994, *SD* = 246 for the blue-light and *M* = 880, *SD* = 204 for the red-light condition. Mean AUCi for cortisol was M = 444, SD = 170 for the bright-light, M = 327, SD = 192 for the dim-light, M = 398, SD = 163 for the blue-light and M = 406, SD = 258 for the red-light condition. AUCg for cortisol was significantly different between the bright- and the dim-light condition, *t* (117) = 4.30, *p* < .001, the bright- and the blue-light condition, *t* (155) = −2.41, *p* = .017, between the dim- and the blue-light condition, *t* (96.7) = −6.45, *p* < .001, between the dim- and the red-light condition, *t* (104) = −2.97, *p* = .004 as well as between the red- and the blue-light condition, *t* (153) = −3.19, *p* = .002, but not between any of the other conditions. AUCi for cortisol proved significantly different between the bright- and the dim-light condition, *t* (174) = 4.25, *p* < .001, the dim- and the blue-light condition, *t* (166) = −2.57, *p* = .011, as well as between the dim- and the red-light condition, *t* (145) = −2.23, *p* = .027, but not between any of the other conditions.

## 4 Discussion

As light plays an important role in circadian rhythmicity (e.g., LeGates et al., 2014) which, in turn, has been observed to influence immune processes (e.g., Haspel et al., 2020; Man et al., 2016; Scheiermann et al., 2013), this preliminary study aimed to investigate the influence of irradiation with different types of light on the anti-inflammatory cytokine IL-10 in healthy adult men. Previous research has provided first evidence of the particular influence of bright and blue light on the stress hormone cortisol (Figueiro and Rea, 2012; Petrowski et al., 2019, 2020a, 2022), which in turn has been observed to influence IL-10 secretion (Cain and Cidlowski 2017; del Giudice and Gangestad, 2018). Additionally, a study by Lewis et al. (2018) reported reduced IL-10 concentrations in post-operative appendicitis patients after exposure to blue light compared to standard room lighting. Against this background, it was hypothesized that bright light and blue light would lead to both increased IL-10 and cortisol concentrations compared to dim light or red light. This was not confirmed in the mixed models. None of the light sources promoted IL-10 or cortisol levels more strongly than the others in this particular sample. Additionally, while IL-10 concentrations significantly changed over time across all light conditions, post-hoc comparisons did not indicate a significant change from one measurement point to the next in any of the light conditions. Upon closer inspection, AUCg measurements for IL-10 seemed to indicate greater total IL-10 output in the red-light condition when compared to the blue-light condition, which would be contrary to expectations. AUCi measurements appeared to show a significantly different IL-10 sensitivity in the bright-light condition when compared to the other conditions and when comparing the red- and blue-light condition to each other. Post-hoc comparisons for cortisol indicated the presence of the CAR in participants. AUCg measurements for cortisol revealed a greater total cortisol output in the bright and blue-light condition, followed by the red-light condition. In all these three conditions total cortisol output greater than in the dim-light condition and cortisol sensitivity differed significantly from it. This is line with previous findings on the influence of light on cortisol. A strength of this preliminary study is the high level of standardization, with participants sleeping at the laboratory and both non-experimental light sources as well as wake-up time being controlled. There were limitations as well: Because of the small sample size, the non-significant findings might be false negative, i.e., significant results might be obtained in larger samples. Additionally, this sample was conducted in a between-subjects design, which restrict causal conclusions when compared to within-subject designs. Regarding IL-10 analysis, the highly-sensitive assays returned a subset of IL-10 values below the detectable level and these we subsequently substituted with a fixed value agreed upon prior. While this has been standard practice (Ge et al., 2024), it can also provide a data set of biased variances that do not reflect what might naturally be observed (Lubin et al., 2004). Because of this, the AUC calculations conducted for IL-10 values have to be taken with a grain of salt, as false positive findings may have resulted from this circumstance. Furthermore, chance introduced some baseline differences between the light condition for some of the cortisol and IL-10 values. Lastly, this preliminary sample only included men.

Future research on this topic should therefore ideally be conducted in a bigger sample more representative of the general population and should take into account the influence on the data set generated by the assays chosen for laboratory analysis. Additionally, in order to gain insight into whether or not IL-10 displays naturally occurring circadian oscillations dependant on light, IL-10 expression should be observed over different and longer time frames during a day as well as in within-subject study designs. Furthermore, different types of light sources and interventions, such as photobiomodulation, should be applied.

More evidence is necessary to arrive at a conclusion as to whether IL-10 concentrations in healthy adults are influenced by either specific light sources or circardian rythmicity in cortisol.

## Funding sources

This research received funding from the 10.13039/501100002347Federal Ministry of Education and Research (10.13039/501100002347BMBF), Germany. This study was conducted in the subproject: “Einfluss von Licht auf die hormonelle Stressverarbeitung” [influence of light on hormonal stress management] as part of the joint project: “Nicht-visuelle Lichtwirkungen” [non-visual effects of light] (Funding code: 13N13397).

## Declaration of generative AI

None.

## Data statement

Due to the sensitive nature of the data, all data presented is available from the corresponding author upon reasonable request only.

## CRediT authorship contribution statement

**Liza Mekschrat:** Writing – review & editing, Writing – original draft, Formal analysis. **Michael Göring:** Data curation. **Bjarne Schmalbach:** Supervision. **Nicolas Rohleder:** Writing – review & editing, Methodology. **Katja Petrowski:** Writing – review & editing, Supervision, Project administration, Funding acquisition.

## Declaration of competing interest

The authors declare the following financial interests/personal relationships which may be considered as potential competing interests:

Katja Petrowski reports financial support was provided by 10.13039/501100002347Federal Ministry of Education and Research Bonn Office Library. If there are other authors, they declare that they have no known competing financial interests or personal relationships that could have appeared to influence the work reported in this paper.

## Data Availability

Data will be made available on request.
